# Downregulation of hippocampal *NR2A/2B* subunits related to cognitive impairment in a pristane-induced lupus *BALB/c* mice

**DOI:** 10.1371/journal.pone.0217190

**Published:** 2019-09-09

**Authors:** Jonatan Luciano-Jaramillo, Flavio Sandoval-García, Mónica Vázquez-Del Mercado, Yanet Karina Gutiérrez-Mercado, Rosa Elena Navarro-Hernández, Erika Aurora Martínez-García, Oscar Pizano-Martínez, Fernanda Isadora Corona-Meraz, Jacinto Bañuelos-Pineda, Jorge Fernando Floresvillar-Mosqueda, Beatriz Teresita Martín-Márquez

**Affiliations:** 1 Universidad de Guadalajara, Centro Universitario de Ciencias de la Salud, Departamento de Biología Molecular y Genómica, Instituto de Investigación en Reumatología y del Sistema Músculo Esquelético (IIRSME), Guadalajara, Jalisco, CP, México; 2 Universidad de Guadalajara, Centro Universitario de Ciencias de la Salud, Departamento de Clínicas Médicas, Guadalajara, Jalisco, CP, México; 3 Universidad de Guadalajara, Envejecimiento, inmuno-metabolismo y estrés oxidativo, Guadalajara, Jalisco, CP, México; 4 Hospital Civil de Guadalajara, Dr. Juan I. Menchaca, División de Medicina Interna, Servicio de Reumatología, Guadalajara, Jalisco, CP, México; 5 Universidad de Guadalajara, Inmunología y Reumatología, Guadalajara, Jalisco, CP, México; 6 Unidad de Evaluación Preclínica, Biotecnología Médica y Farmacéutica, CONACYT Centro de Investigación y Asistencia en Tecnología y Diseño del Estado de Jalisco (CIATEJ), Guadalajara, CP, México; 7 Universidad de Guadalajara, Centro Universitario de Tonalá, Departamento de Ciencias Biomédicas, División de Ciencias de la Salud, Tonalá, Jalisco, CP, México; 8 Universidad de Guadalajara, Centro Universitario de Ciencias Biológicas y Agropecuarias, Departamento de Medicina Veterinaria, Zapopan, Jalisco, CP, México; 9 Universidad de Guadalajara, Centro Universitario de Ciencias de la Salud, Departamento de Microbiología, Guadalajara, Jalisco, CP, México; Technion Israel Institute of Technology, ISRAEL

## Abstract

Neuropsychiatric systemic lupus erythematosus (NPSLE) is associated with learning and memory deficit. Murine model of lupus induced by pristane in *BALB/c* mice is an experimental model that resembles some clinical and immunological SLE pathogenesis. Nevertheless, there is no experimental evidence that relates this model to cognitive dysfunction associated with *NR2A/2B* relative expression. To evaluate cognitive impairment related to memory deficits in a murine model of lupus induced by pristane in *BALB/c* mice related to mRNA relative expression levels of *NR2A/2B* hippocampal subunits in short and long-term memory task at 7 and 12 weeks after LPS exposition in a behavioral test with the use of Barnes maze. A total of 54 female *BALB/c* mice 8–12 weeks old were included into 3 groups: 7 and 12 weeks using pristane alone (0.5 mL of pristane) by a single intraperitoneal (i.p.) injection. A control group (single i.p. injection of 0.5 mL NaCl 0.9%) and pristane plus LPS exposure using single i.p. pristane injection and LPS of *E*. *coli* O55:B5, in a dose of 3mg/kg diluted in NaCl 0.9% 16 weeks post-pristane administration. To determine cognitive dysfunction, mice were tested in a Barnes maze. Serum anti-Sm antibodies and relative expression of hippocampal *NR2A/2B* subunits (*GAPDH* as housekeeping gene) with SYBR green quantitative reverse transcription polymerase chain reaction and 2^-ΔΔC^_T_ method were determined in the groups. Downregulation of hippocampal *NR2A* subunit was more evident than *NR2B* in pristane and pristane+LPS at 7 and 12 weeks of treatment and it is related to learning and memory disturbance assayed by Barnes maze. This is the first report using the murine model of lupus induced by pristane that analyzes the NMDA subunit receptors, finding a downregulation of *NR2A* subunit related to learning and memory disturbance being more evident when they were exposed to LPS.

## Introduction

Systemic lupus erythematosus (SLE) is an idiopathic autoimmune disorder characterized by the induction of autoantibodies against intracellular components such as nucleosomes, double-stranded DNA and histones, and small nuclear ribonucleoproteins (snRNPs) the more important (pathognomonic) known as Smith antigen (anti-Sm), that is consider for the American College of Rheumatology (ACR) as a classification criteria for SLE diagnosis [1, 2]. This condition presents a wide variety of clinical manifestations with multiple-organ affectations, especially skin and kidneys, however the heart and central and/or pheripheral nervous system are also implicated[3, 4]. In 1999, the ACR established a standard nomenclature with case definitions for 19 neuropsychiatric conditions, 12 related to central nervous system (CNS) manifestations (mainly seizures, headache, stroke, depression, cognitive dysfunction, and psychosis)[3, 5]. Clinical studies estimate a prevalence of neuropsiquiatric lupus (NPSLE) from 17 to 80%, these variations can be attributed to diagnostic criteria and patient selection[3, 6–8]. The etiopathogenesis of NPSLE is still unknown, however several studies suggest that the presence of autoantibodies against to N-methyl-D-aspartate (NMDA) receptors in serum and cerebrospinal fluid (CSF) might be the result of production of intrathecal proinflammatory cytokines/chemokines and vasculitis[3–5]. Studies in human SLE patients and murine model of lupus report that anti-double-stranded DNA (anti-dsDNA) and anti-NMDA could migrate from the peripheral blood to the central nervous system through a disrupted blood-brain barrier (BBB). Anti-dsDNA might cross-react with a consensus pentapeptide (DWEYS) present in NR2A and NR2B subunits of NMDA receptors, mediating neuronal loss with the consequence of a deficit in learning and memory process[4, 9–11].

In order to induce some clinical manifestations that resemble NPSLE, the experimental murine models of lupus must gather two requirements: the production of autoantibodies that cross-react with neural receptors and the disruption of BBB by exposure to lipopolysaccharide (LPS)[4]. Lupus can be induced by exposing a wild type mouse strain (*BALB/c*) to hydrocarbon oils such as pristane (2,6,10,14-tetramethylpentadecane) that generates a wide range of specific SLE autoantibodies (anti-dsDNA, anti-RNP/Sm and anti-Su) between 12 to 25 weeks after induction with 0.5 mL of intraperitoneal injection of pristane[12–14]. This is a suitable model to evaluate the break of immune tolerance induced by environmental factors associated with murine lupus development. Nevertheless, there is no experimental evidence in murine model of lupus induced by pristane about cognitive dysfunction associated with the development of autoantibodies against hippocampal NMDA receptor subunits NR2A/2B.

To evaluate cognitive impairment related to memory deficits in a murine model of lupus induced by pristane (*BALB/c* strain), we analyzed the mRNA expression levels of *NR2A/2B* hippocampal subunits in a short and long-term memory tasks at 7 and 12 weeks after LPS exposure assessing the behavior using Barnes maze.

## Materials and methods

### Animals

Female *BALB/c* mice 8–12 weeks old were obtained from UNAM-Envigo RMS Laboratory in Mexico City. They were housed in the animal facility of the Instituto de Investigación en Reumatología y del Sistema Músculo Esquelético, Centro Universitario de Ciencias de la Salud, Universidad de Guadalajara, under the following conditions: 2–4 animals in clear cages (7.6x11.6x4.8 inches). The room temperature was controlled at 22±1°C, positive laminar flow, 12 hours of light/dark cycles and the mice were fed *ad libitum* with purified water and normocaloric diet (Rodent Chow 5001, Purina^TM^). The protocol was approved by the Committee of research, ethics and biosecurity of the Centro Universitario de Ciencias de la Salud, Universidad de Guadalajara (Protocol number CI-07918) and all experimental procedures were carried out in compliance with the rules for research in health matters (NOM 0062-ZOO-1999 and NOM-033-ZOO-1995).

#### Murine model of lupus induced by pristane

**Fig 1:**
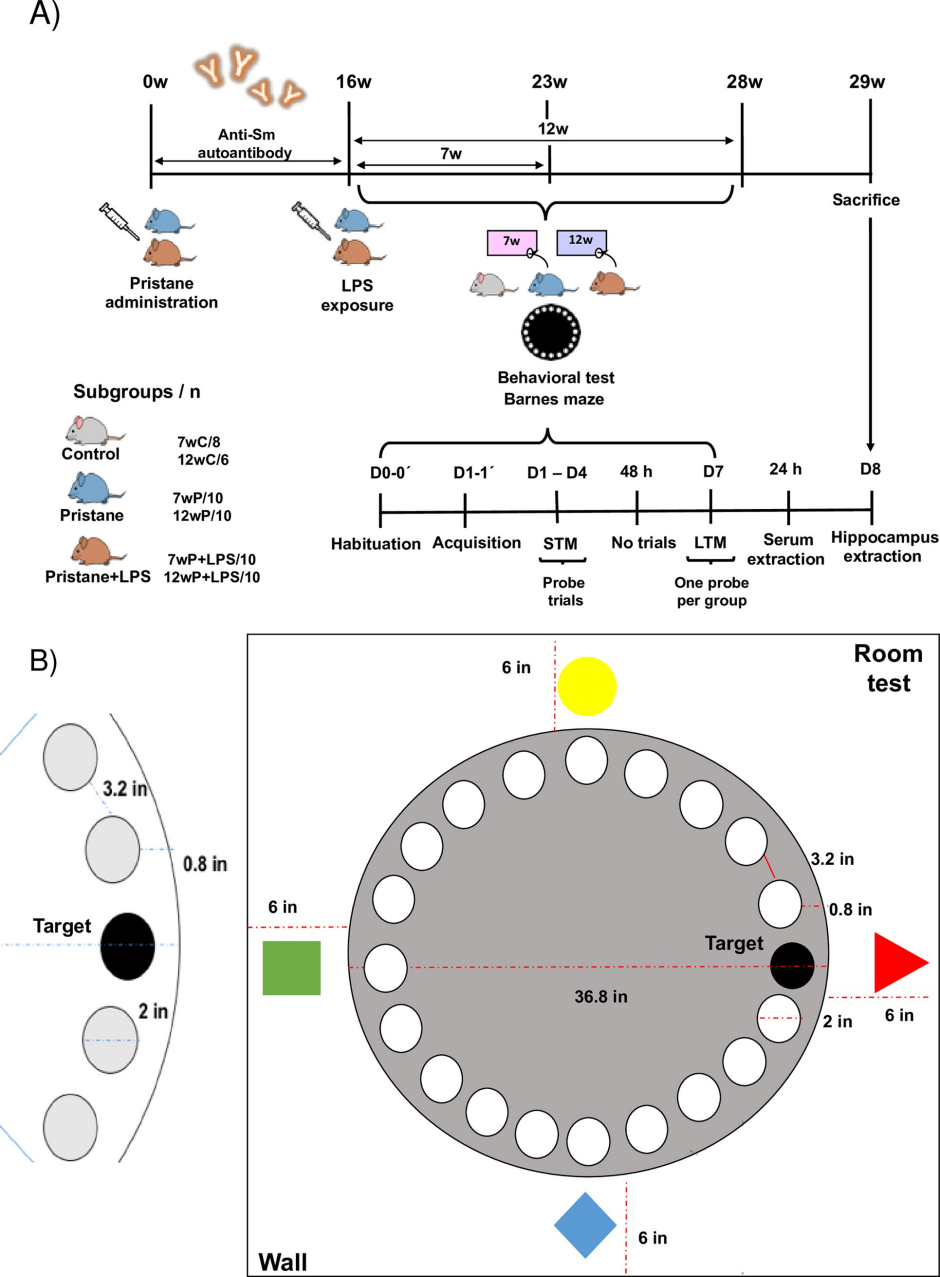
Eschematic representation of the experimental procedures in tested groups and Barnes maze test. A) Time point experimental procedures, behavioral test using Barnes maze and B) Eschematic design of Barnes maze platform. Abbreviatures: w = weeks, C = control, P = Pristane, P+LPS = Pristane plus LPS, D = day, h = hours, STM = short-term memory, LTM = long-term memory, in = inches.

A total of 54 female *BALB/c* mice 8–12 weeks old were included into 3 groups: 7 and 12 weeks using pristane (P) alone (7wP and 12wP, 10 mice each, 0.5 mL of pristane (Sigma Chemical Co, St Louis, MO, USA) by a single intraperitoneal (i.p.) injection and a control group (7wC and 12wC, 8 and 6 respectively) by a single intraperitoneal injection (i.p.) of 0.5 mL NaCl 0.9%) (Fig 1A).

#### Blood brain barrier disruption with LPS in a murine model of lupus induced by pristane

Pristane plus LPS exposure (P+LPS, 10 mice each, 7wP+LPS and 12w P+LPS) using single i.p. pristane injection and LPS of *E*. *coli* O55:B5, Sigma St Louis, MO, USA in a dose of 3mg/kg diluted in NaCl 0.9% 16 weeks post-pristane administration[15].

#### Barnes maze

To determine cognitive dysfunction, we evaluated memory and learning process with Barnes maze test in the experimental groups at 7 and 12 weeks after BBB disruption with LPS in a murine model of lupus induce by pristane[16]. The behavioral Barnes test adapted for mice consisted in a circular black acrylic platform of 36.8 inches of diameter anchored in a metallic base of 32 inches of height above the floor. The platform has 20 holes of 2 inches of diameter disposed along the perimeter with 3.2 inches among them (19 empty holes and 1 escape hole with dark box). As reference points to reach the escape hole, we used extra-maze cues around the room (yellow circle, blue rhombus, red triangle, and green square). To eliminate odor cues, the experimenter cleaned the platform and the escape box after every trial with 70% ethyl alcohol (Fig 1B). To assess the memory and learning process, we decided to record each mouse studied using the Pro Webcam-C920 HD 1080p. Afterwards, two observers (one blinded), scored manually the behavior (latency and errors) of each tested animal[17].

#### Behavioral test

The behavioral test was developed by two experimenters in three phases: habituation, acquisition and probe trial assessed in an airy and odor free white room without visual and sound distractors. The habituation phase consisted in two days (Day 0 and Day 0’) with two trials per mouse in the platform and escape hole with a time lapse of 180 seconds. In the acquisition phase (Day 1 and Day 1’) we considered two trials per mouse. It consisted in placing the mouse into a white acrylic cylindrical chamber in the middle of the platform during 10 seconds and then release it to explore the platform for a time elapse of 180 seconds finishing the trial when the mouse entered by itself into the escape hole. In this phase, if the mouse did not enter the target hole, the experimenter could guide it. The probe test trials were assessed to evaluate memory and learning consolidation in all mice groups tested and consisted in 3 repetitions per mouse on the maze to reach the escape hole in 180 seconds, taking into account all the procedures described previously. This phase was denominated Short Term Memory (STM) and consisted of four days probe trials (D1-D4). Once finished, the mice had no probe trials for 48 hours and then were re-evaluated for the Long Term Memory (LTM) at day 7 (D7). We considered to evaluate the behavioral performance with two parameters: the escape latency in seconds to reach and enter the escape hole and the total errors established as the deflections of the head in empty holes for each mouse prior to finding and entering the escape hole (Fig 1A).

#### Assay of anti-Sm antibodies and proteinuria

Once the behavioral tests were finished, we obtained around 300–400 microliters of whole blood extracted per mouse from tail vein, next centrifuged at 3,500 RPM for 10 minutes and the serum was separated and stored at -20°C. A total of 100 to 200 microliters of serum was obtained, for the enzyme-linked immunosorbent assay (ELISA) assay using 1:2 dilution for the detection of anti-Sm antibodies. Levels of mouse anti-Sm antibodies in sera from experimental groups were assayed using the quantitative kit Mouse Anti-Sm Ig’s (total/A+G+M) (Alpha Diagnostic International^TM^). At the same time, the proteinuria was determinated with Multistix^™^ 10 SG (Bayer ^™^).

#### RNA isolation and *NR2A/2B* subunits by qRT-PCR analysis

Once finishing the behavioral test, the mice were euthanized by CO_2_ inhalation and after craniotomy surgery; the hippocampus region was removed to obtain lysates for total RNA isolation. This procedure was performed according to the manufacturer’s procedure using the GF-1 Total RNA extraction kit (Vivantis Technologies^TM^). The complementary DNA synthesis (cDNA) was performed with 5μg of each total RNA sample using a reaction size of 20μL with oligo (dT) primer (100 ng/μL), RNase free, DEPC-treated water and Moloney Murine Leukemia Virus Reverse Transcriptase (M-MLV RT) kit (Applied Biosystems, 850 Lincoln Centre Drive, Foster City, CA 94404) and stored at -20°C until being used for expression analysis. Real-time quantitative polymerase chain reaction (qRT-PCR) was conducted using Rotor-Gen (Q5 PLEX HRM System, Qiagen^TM^). A threshold cycle (C_T_) value was determined from each amplification plot. For *Mus musculus* genes, the specific primers were synthesized based on sequences published by Hamada *et al*.[18] as follows: *GluN2A* forward 5´-CCTTTGTGGAGACAGGAATCA-3´ and reverse 5´-AGAGGCGCTGAAGGGTTC-3´; *GluN2B* forward 5´-GGGTTACAACCGGTGCCTA-3´ and reverse, 5´-CTTTGCCGATGGTGAAAGAT-3´. The expression of target genes was normalized with the endogenous reference mouse gene *GAPDH* using the following primers: forward 5´-TGTCCGTCGTGGATCTGAC-3´ and reverse 5´-CCTGCTTCACCACCTTCTTG-3´. The qPCR was performed in a final reaction volume of 10μL (10μM forward and reverse primer, 25μM ROX, 2x SYBR Green qPCR master mix and 100ng cDNA). The conditions of the reaction were: holding at 95°C/10 min, cycling at 35 cycles of 95°C/10s and 55°C/15s and melt curve at 95°C/15s, 72°C/60s, and 95°C/15s.

#### Statistical analysis

We used the Kolmogorov-Smirnov test to determine the distribution of the data. Since we considered that only non-parametric test could be used, comparisons were made using Kruskal-Wallis employing Mann-Whitney U as applicable. Values are presented as mean and standard deviation and as mean and standard error of mean (±SEM), as applicable. Spearman’s correlations coefficients were also calculated. All data were analyzed using SPSS v22.0 (SPSS Inc. Chicago, IL) and GraphPad Prism version 6.00 for Windows (GraphPad Software, La Jolla, CA). *P* <0.05 was considered statistically significant.

## Results

### Escape latency in the STM and LTM phase at 7 weeks in a murine model of lupus induced by pristane

**Fig 2:**
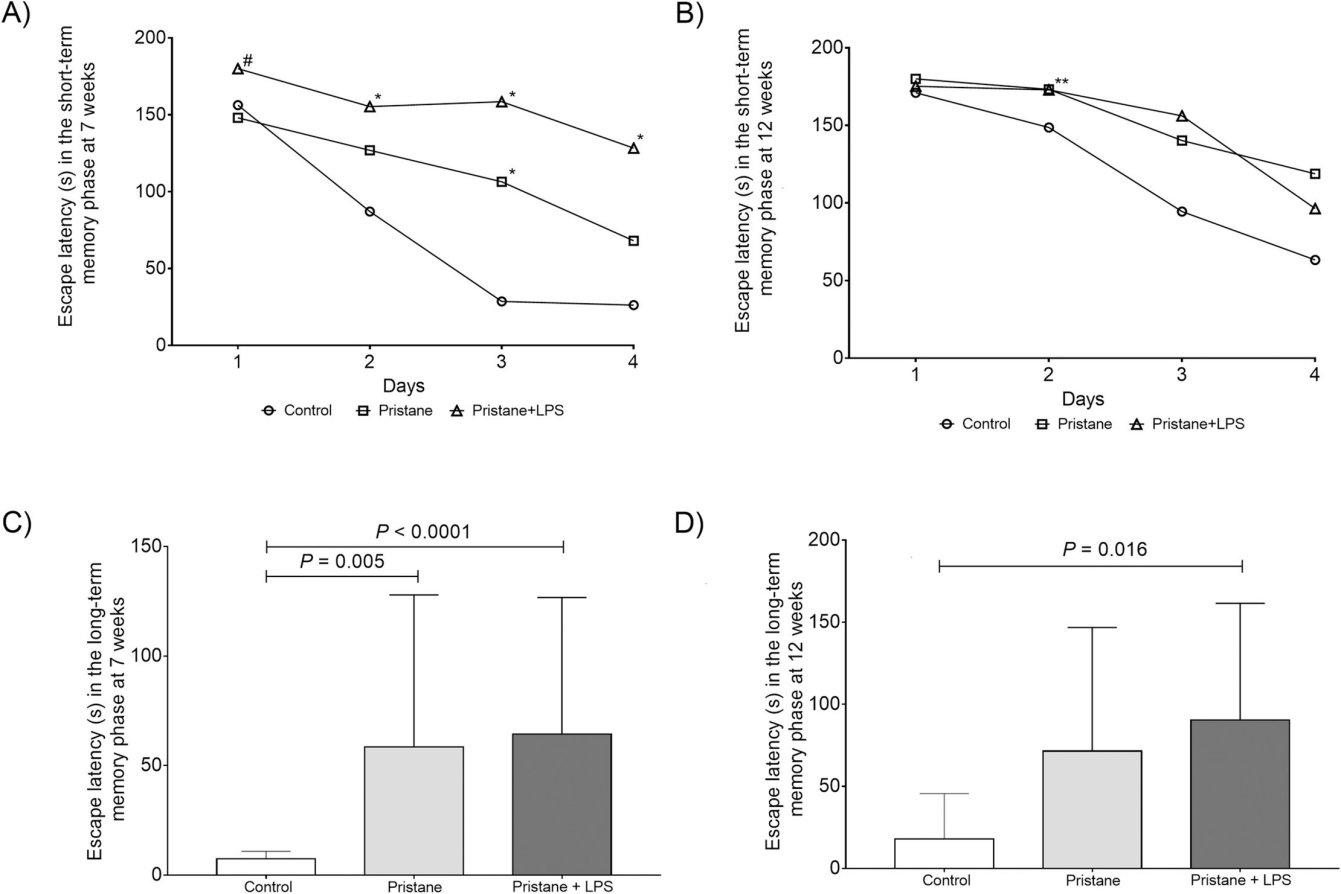
Escape latency of experimental subgroups in STM and LTM. Data are shown in $\bar{x}$ ± SE. #differences between pristane and pristane+LPS groups, *differences between control and pristane or pristane+LPS. Unpaired Mann Whitney U test, *P* < 0.05. A) Escape latency in the STM phase at 7 weeks. B) Escape latency in the STM memory phase at 12 weeks. C) Escape latency in the LTM phase at 7 weeks. D) Escape latency in the LTM phase at 12 weeks.

Once the mice completed the habituation and acquisition probes in Barnes maze, we evaluated the STM in all the groups tested (control, pristane and pristane+LPS) at 7 weeks during D1-D4 in the exploration time to reach the target hole and enter the escape hole and we found the following statistical differences. In day 1: between pristane 162.2s (120-180s) *vs*. pristane+LPS 180s (179.9-180s, *P* = 0.033). In day 2: between control 97.3s (34.7–133.6s) *vs*. pristane+LPS (113-180s, *P =* 0.009). In day 3: between control 22.1s (14.9–30.1s) *vs*. pristane 125s (33.3-180s, *P* = 0.014) and control *vs*. pristane+LPS 145.6s (116.5–152.8s, *P*<0.0001) and, in day 4: between control 12.2s (7.0–39.1s) *vs*. pristane+LPS 175s (40.3-180s, *P* = 0.003) (Fig 2A).

We want to highlight the pristane+LPS were the most affected among all groups tested. In addition, when we evaluated LTM 48 hours we observed a prolonged escape latency between control *vs*. pristane (6s *vs*. 14s, *P* = 0.005) and control *vs*. pristane+LPS (6s *vs*. 35s, *P*<0.0001). In this probe, the total of control mice reached the target hole in less time in comparison to the mice treated with pristane and pristane+LPS (Fig 2C).

### Escape latency in the STM and LTM phase at 12 weeks in a murine model of lupus induce by pristane

With the purpose of determinig if the induction of lupus by pristane and the exposure to LPS maintain the cognition deficit in a prolonged manner, we used the same experimental groups and evaluated them at 12 weeks (Fig 2B and 2D). Comparing the results described previously at 7 weeks at this time point (12 weeks) we observed only a statistical difference in escape latency in day 2 between control *vs*. pristane (141.5s *vs*. 180s, *P* = 0.013) and control *vs*. pristane+LPS (141.5s *vs*. 180s 171.5–180, *P* = 0.013).

We evaluated the escape latency in LTM 12 weeks (Fig 2D) the observed difference was found between control *vs*. pristane+LPS (11s *vs*. 140s, *P* = 0.016).

### Total errors in the STM and LTM phase at 7 and 12 weeks

At the same time of STM evaluation, we calculated the number of errors between groups in D1-D4 and we observed differences only in day 4 between control 0.5 (0–1) *vs*. pristane 3 (2–6, *P* = 0.04) at 7 weeks.We did not observe differences in errors between the subgroups during D1-D4 in STM at 12 weeks, as well as LTM at 7 and 12 weeks (S1 Fig https://figshare.com/articles/Supporting_information/9722192).

### Validation of murine model of lupus induced by pristane assayed by the presence of anti-Sm antibodies and proteinuria

We quantified the serum levels of anti-Sm antibodies in experimental groups by ELISA at 7 and 12 weeks. In 7 weeks the results were as follows: control group 702.6 ± 418.0 U/mL, pristane 1453.7 ± 743.3 U/mL and pristane+LPS 995.4 ± 375.0 U/mL (736.6–2095 U/mL).

In 12 weeks we observed the following results: control group 571.0 ± 277.6 U/mL, pristane 2457.9 ± 1607.9 U/mL and pristane+LPS 2901.8 ± 2163.2 U/mL.

**Fig 3:**
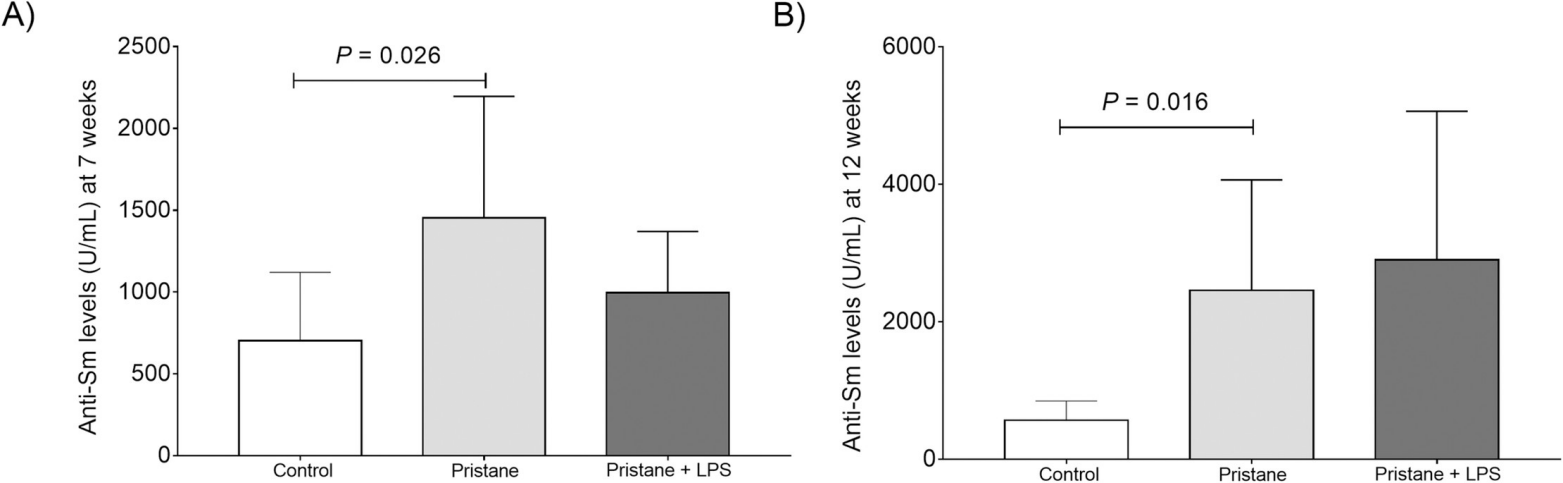
Serum levels of anti-Sm antibodies at 7 and 12 weeks. Data are shown in $\bar{x}$ ± SE. Differences between groups unpaired Mann Whitney U test, *P* < 0.05. A) Anti-Sm antibodies serum levels at 7 weeks. B) Anti-Sm antibodies serum levels at 12 weeks.

We observed statistical differences between the control group *vs*. pristane in 7 and 12 weeks (Fig 3).

Significant proteinuria was detected in more than 30 mg/dL in 8% of control group, 80% in pristane and 60% pristane+LPS. Proteinuria above a 100 mg/dL was found in 0% in control, 0% pristane and 33% in pristane+LPS groups (S2 Fig https://figshare.com/articles/Supporting_information/9722192).

### *NR2A* subunit expression was downregulated in a murine model of lupus induced by pristane and pristane plus LPS at 7 and 12 weeks

We observed in the pristane group 55% less expression (P = 0.002) than control group at *NR2A* subunit at 7 weeks being more evident in 93% less expression (P = 0.016) than control group at 12 weeks.

**Fig 4:**
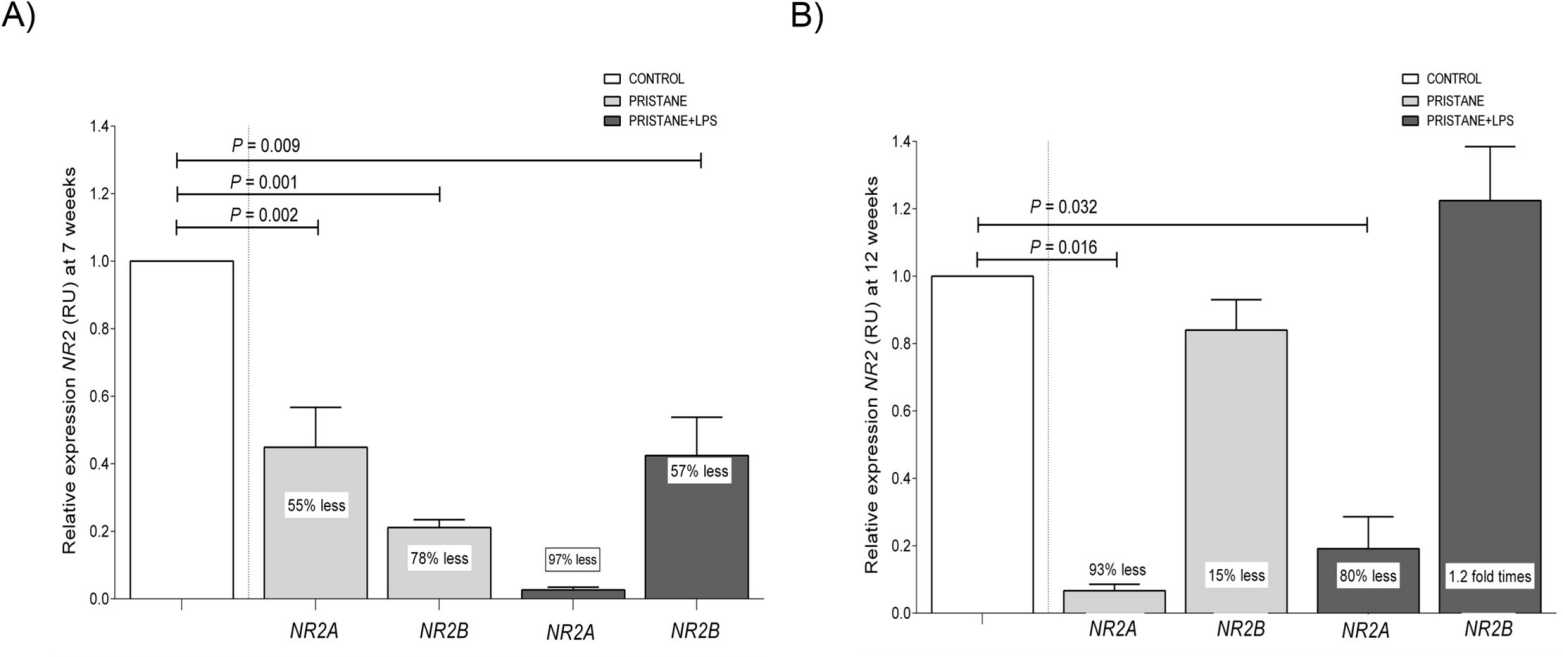
*NR2A/2B* subunit expression of the murine hippocampus at 7 and 12 weeks. Data are shown in $\bar{x}$ ± MSE, differences between groups unpaired Mann Whitney U test, *P* < 0.05. A) Relative expression of *NR2A* and *NR2B* at 7 weeks. B) Relative expression of *NR2A* and *NR2B* at 12 weeks.

Besides in the pristane+LPS treated group, we were able to demonstrate 80% less expression (P = 0.032) than control group at 12 weeks (Fig 4A and 4B).

### *NR2B* subunit expression was downregulated in a murine model of lupus induced by pristane and pristane plus LPS at 7 weeks

We observed in the pristane treated group 78% less expression (*P* = 0.001) than control group at *NR2B* subunit at 7 weeks. In contrast we showed 57% less expression than the control (*P* = 0.009) in pristane+LPS at 7 weeks (Fig 4A and 4B).

Also, *NR2B* subunit expression correlates negatively with the levels of anti-Sm antibodies in pristane and pristane+LPS in 7 and 12 weeks (S3 Fig https://figshare.com/articles/Supporting_information/9722192).

## Discussion

NPSLE is considered a severe condition of SLE physiopathology and cognitive dysfunction is the more frequent neuropsychiatric alteration with a 15–81% of prevalence [19]. Several studies have proposed that autoantibodies such as anti-dsDNA and anti-Sm produce a cross-reaction with neuronal receptors, attributing a potential pathogenic role in NPSLE[20–22]. One description of this hypothesis was published by Bluestein *et al*. in 1981 where they demonstrated an increased immunoglobulin G (IgG) antineural activity in CSF in SLE patients with active CNS manifestations[23]. These results are in accordance with the analysis performed by How *et al*. in 1985, who demonstrated an association between serum antineuronal autoantibodies and NPSLE manifestations [24].

Nowadays, cognitive dysfunction in NPSLE is associated with the presence in serum and CSF of antiphospholipid antibodies and anti-NMDA receptor subunit NR2A (anti-NR2A) antibodies levels, in addition to disease activity, glucocorticoids use, hypertension [3, 19, 25], and seizures [26]. Besides other components such as NR1 subunit specifically some aminoacids residues of the N-terminous might play a role in NPSLE [27]. Since is not ethical neither possible to do experimental interventions in order to see the possible presence of autoantibodies within the brain tissue in human SLE patients, the use of murine models are a valuable tool to develop experimental assays to go further in the knowledge of NPSLE such as induction of excitotoxic neuronal death associated to cognitive impairment in the hippocampal region [15]. In our study, we decided to explore the possible cognitive impairment in a murine model of lupus induce by pristane. The pristane stimulates in female *BALB/c* mice the production of proinflammatory cytokines and autoantibodies such as anti-Sm, anti-dsDNA, and anti-U1RNP that in addition to LPS, we hipothetized that could be a disruption in the BBB[28]. This phenomenon has been shown in SLE patients through magnetic resonance imaging (MRI) corroborating high levels of permeability of BBB, particularly in hippocampus region related to autoantibody production [29].

Our study was proposed with the aim of determining cognitive impairment because it had not been performed in this model before. With the purpose of evaluating the learning and memory process in murine models, behavioral tests are used to assess hippocampal deterioration associated with neuropathologic alterations. The strategic test used to analyze cognitive performance in mice is the Barnes maze, which consists of an elevated circular platform with empty holes and one escape hole around the perimeter. This test takes advantage of the natural preference of rodents for the dark environment and is not influenced by hunger motivation neither stress [30].

The Barnes maze and Morris water maze are two commonly-used tests of spatial memory, of which the water maze is considered more stressful. Water-maze training induced a greater increase in plasma cortisol than did Barnes maze. Importantly, spatial learning was inversely correlated with cortisol levels in the water maze but not in the Barnes maze, suggesting that the performance of water maze migth be more stressful even in wild-type mice of the same age and gender [30, 31]. In this protocol, we used the Barnes maze test in a murine model of lupus induced by pristane in *BALB/*c mice divided in a control group, and two experimental ones (pristane and pristane+LPS at 7 at 12 weeks). We evaluated the STM and LTM in the described groups. The Barnes maze protocol considers two basic parameters: the escape latency and errors, both of them evaluated in our study during STM and LTM during STM (D1-D4) and LTM (D7). In the STM at 7 weeks trial, we observed significant differences in escape latency between pristane/pristane+LPS *vs*. control group in day 3 and day 4 (D3, D4) and maintained in LTM. In the control mice, as consequence of memory consolidation and learning process we observed less escape latency as expected which correlates with other reported studies [17]. Nevertheless, at 7 and 12 weeks of experimentation, mice treated with pristane and pristane+LPS showed an unexpected behavior and freezing attitude (no movement) that resulted in a prolonged time to reach and enter the escape hole, so we were unable to observe errors during the time elapsed.

In our study, we quantified the hippocampal mRNA expression levels of *NR2A* and *NR2B* by qRT-PCR and we observed a downregulation in the relative expression of *NR2A* at 7 and 12 weeks with a remarkable reduction in pristane+LPS group, we could suggest that aging process itself, is not responsable for NR2A subunit downregulation, since it is clear that control mice did not change the mRNA expression neither at the same time points.

Pristane treatment alone seems to downregulates NR2A but in a slow manner *vs*. pristane plus LPS, being evident since 7 weeks until the end of the experiment. The remarkably downregulation of *NR2A* might be the result of a synergic action between LPS and pristane treatment previously no reported.

Notwithstanding, LPS increase the blood brain barrier permeability. In this context, it is recognize that LPS itself is able to cause brain damage through receptor Toll-like receptor-4 (TLR4) pathway proinflammatory cytokines, nitric oxide and prostaglandin 2 (PGE2) as a consequence an upregulation effect is observed in the mRNA *NR2B* levels[32] (Fig 2A and 2B).

Regarding neuronal development and memory consolidation in rodents, studies confirm that the NR2A/2B subunits of NMDA mediate certain forms of synaptic plasticity and learning. These receptors are differentially expressed over development with NR2B predominance in mouse brain until NR2A expression increases from the second postnatal week influenced by learning and sensory experience [33]. Successful olfactory discrimination learning in rats is associated with an increase in the NR2A/NR2B ratio and has been proposed that the increase in NR2A estabilizes memory [34] and is associated with the emotional behavior regulation in mice [35]. On the other hand, in relation to anti-Sm antibodies and neuronal receptors, it has been demonstrated that these autoantibodies can disrupt BBB and have a potential neurotoxic effect that is considered a prognostic factor for acute confusional state (ACS) in SLE [21, 22]. However, more evidence is needed to determine the biological role of an autoantibody such as anti-Sm (pathognomonic but not pathogenic in human SLE) in the context of antibodies against NR2A/2B subunit receptor in murine lupus cognitive impairment.

These results show at 7 weeks (23 weeks of murine lupus Fig 1A), that pristane might have the potential to induce an anti-Sm antibody production, that could have a potential role in the disruption of the BBB that in presence of LPS, these effects could act in a synergistic manner with the production of proinflammatory cytokines [36].

## Conclusions

This is the first report using the murine model of lupus induced by pristane that analyzes the NMDA subunit receptors, finding a downregulation of *NR2A* subunit related to learning and memory disturbance being more evident when they were exposed to LPS.

## Supporting information

S1 FigTotal errors of experimental subgroups in STM and LTM.Data are shown in $\bar{x}$ ± SE. *differences between control and pristane or pristane+LPS groups. Unpaired Mann Whitney U test, *P* < 0.05. A) Total errors in the short-term memory phase at 7 weeks. B) Total errors in the STM phase at 12 weeks. C) Total errors in the LTM phase at 7 weeks. D) Total errors in the long-term memory phase at 12 weeks(TIF)

S2 Fig Proteinuria frequency in the groups. Data are shown in percentage.
(TIF)

S3 Fig Correlation between anti-Sm antibodies levels and *NR2A/NR2B* subunit expression. **Figure A-D** A) Anti-Sm antibodies levels/mRNA expression levels of *NR2A* at 7 weeks. B) Anti-Sm antibodies levels/mRNA expression levels of *NR2A* at 12 weeks. C) Anti-Sm antibodies levels/mRNA expression levels of *NR2B* at 7 weeks. D) Anti-Sm antibodies levels/mRNA expression levels of *NR2B* at 12 weeks. Spearman rho correlations.
(TIF)
