# Supplementary figures and images for: Downregulation of hippocampal NR2A/2B subunits related to cognitive impairment in a pristane-induced lupus BALB/c mice
Source: PLoS One. 2019 Sep 9;14(9):e0217190. doi: 10.1371/journal.pone.0217190 (PMC6733477; doi:10.1371/journal.pone.0217190)

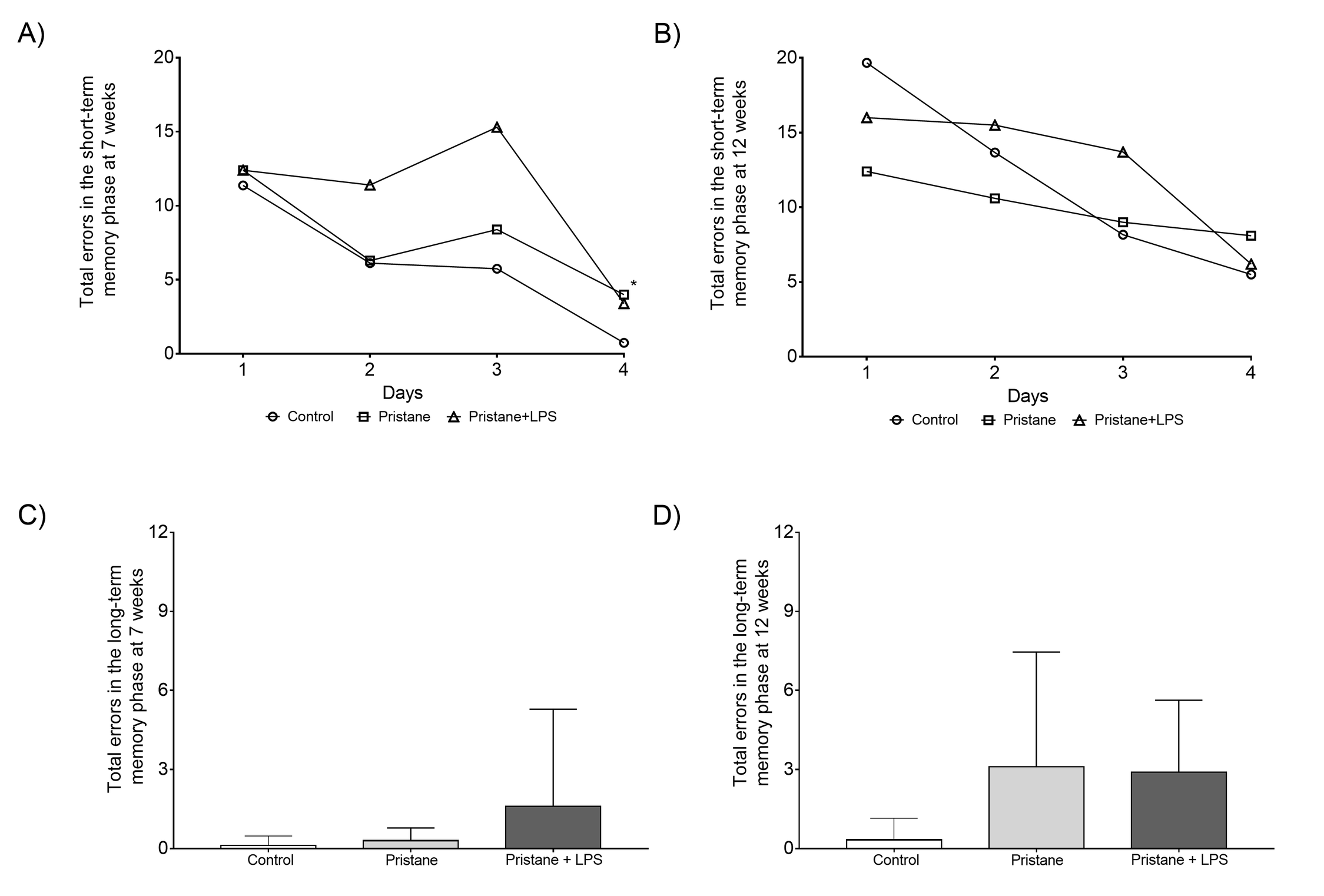

Supplement: S1 Fig — Data are shown in x¯ ± SE. *differences between control and pristane or pristane+LPS groups. Unpaired Mann Whitney U test, P < 0.05. A) Total errors in the short-term memory phase at 7 weeks. B) Total errors in the STM phase at 12 weeks. C) Total errors in the LTM phase at 7 weeks. D) Total errors in the long-term memory phase at 12 weeks (TIF) [file pone.0217190.s001.tif]

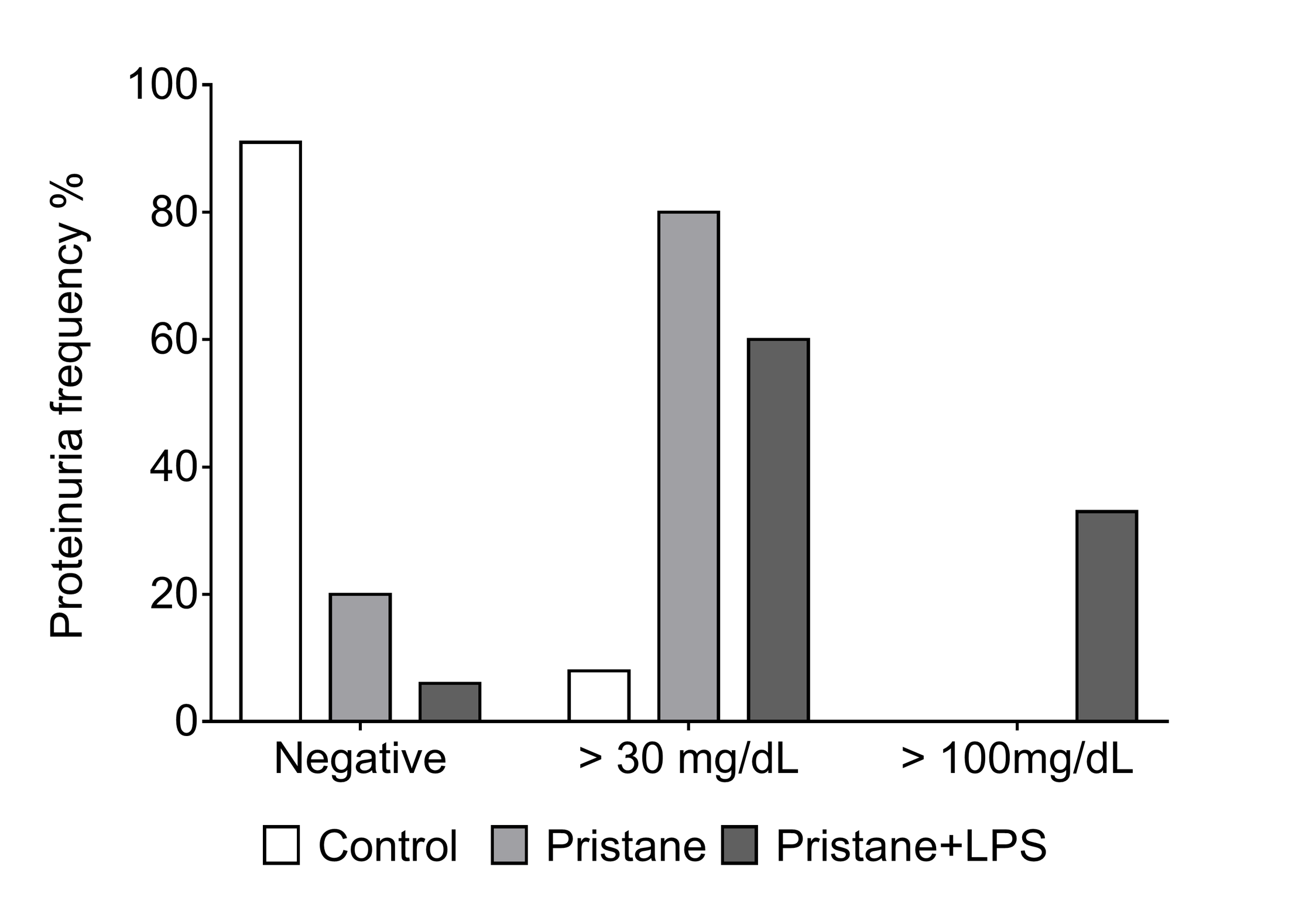

Supplement: S2 Fig — Data are shown in percentage. (TIF) [file pone.0217190.s002.tif]

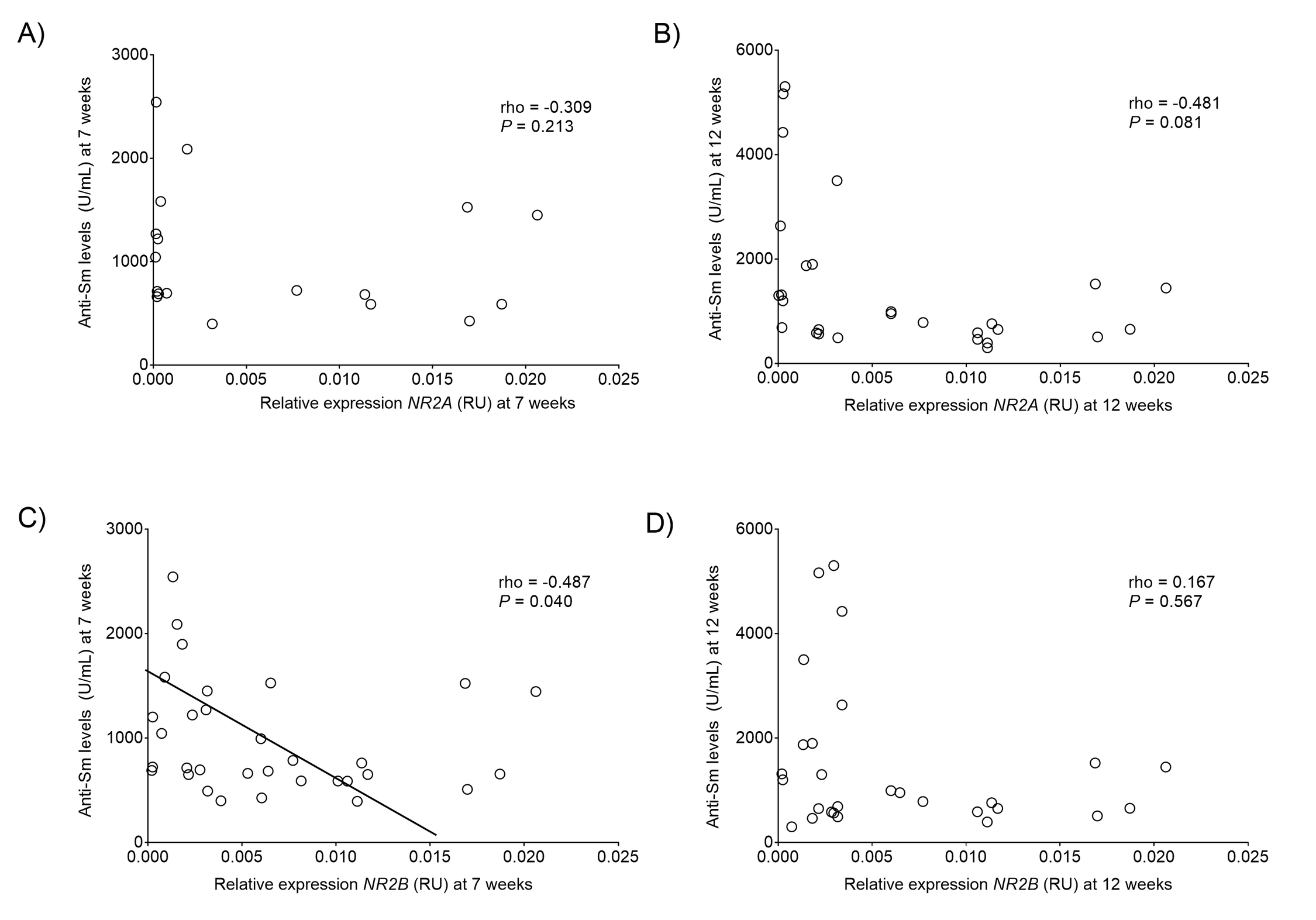

Supplement: S3 Fig — Figure A-D A) Anti-Sm antibodies levels/mRNA expression levels of NR2A at 7 weeks. B) Anti-Sm antibodies levels/mRNA expression levels of NR2A at 12 weeks. C) Anti-Sm antibodies levels/mRNA expression levels of NR2B at 7 weeks. D) Anti-Sm antibodies levels/mRNA expression levels of NR2B at 12 weeks. Spearman rho correlations. (TIF) [file pone.0217190.s003.tif]
